# Overlap of membranous nephropathy and IgA nephropathy in a patient with Kimura’s disease: a case report and literature review

**DOI:** 10.3389/fimmu.2024.1404954

**Published:** 2024-07-12

**Authors:** Géssica Sabrine Braga Barbosa, Precil Diego Miranda de Menezes Neves, Sara Mohrbacher, André Néder Ramires Abdo, Lívia Barreira Cavalcante, Yara de Menezes, Victor Augusto Hamamoto Sato, Érico de Souza Oliveira, Leonardo Victor Barbosa Pereira, Alessandra Martins Bales, Marcella Martins Frediani, Pedro Renato Chocair, Américo Lourenço Cuvello-Neto

**Affiliations:** ^1^ Nephrology and Dialysis Center, Oswaldo Cruz German Hospital, São Paulo, Brazil; ^2^ Nephrology Division, University of São Paulo School of Medicine, São Paulo, Brazil; ^3^ Internal Medicine Service, Oswaldo Cruz German Hospital, São Paulo, Brazil; ^4^ Hematology Service, Oswaldo Cruz German Hospital, São Paulo, Brazil; ^5^ Pathology Service, Oswaldo Cruz German Hospital, São Paulo, Brazil

**Keywords:** Kimura’s disease, nephrotic syndrome, membranous nephropathy, IgA nephropathy, kidney biopsy

## Abstract

**Introduction:**

Kimura’s disease (KD) is a rare chronic inflammatory disorder characterized by subcutaneous lymphoid hyperplasia with peripheral eosinophilia. Kidney involvement is reported in 15%–18% of adult patients with KD, in many cases as nephrotic syndrome. We present a case of overlapping membranous nephropathy and IgA nephropathy associated with KD.

**Case report:**

A 27-year-old man was admitted with a history of bilateral leg edema for the last 2 months and concomitant progressive increase of cervical mass and fever. Laboratory findings were as follows: peripheral leukocyte count, 10,080/mm³; eosinophils, 3,200/mm³ (31.7%); serum creatinine, 0.83 mg/dL; and eGFR: 140 mL/min per 1.73 m^2^. Urinalysis revealed the presence of hematuria and proteinuria and the following results: 24-h proteinuria, 12.9 g; serum albumin, 1.3 g/dL; and elevated IgE level, 750 kU/L. Serologies for hepatitis B, hepatitis C, HIV, and VDRL were all negative. Complement C3 and C4 levels were normal. No monoclonal protein was detected in blood and urine. Parasite infestation was discarded. A biopsy of the cervical lymph node revealed eosinophilic lymphoid hyperplasia, suggesting KD. A kidney biopsy revealed findings consistent with the overlapping of membranous nephropathy with IgA nephropathy. The patient was treated for KD with prednisone 1 mg/kg/d with progressive dose tapering and posterior association of methotrexate 15 mg/week. A renin–angiotensin system inhibitor was prescribed for nephrotic syndrome. The cervical mass regressed, and proteinuria achieved partial remission, with an increase in serum albumin level and normalization of eosinophils and IgE levels.

**Conclusion:**

Although uncommon, kidney involvement must be considered in patients with KD. Glomerular diseases are the most frequent form of kidney injury.

## Introduction

Kimura’s disease (KD) is a rare chronic inflammatory disorder characterized by lymphoid hyperplasia with peripheral eosinophilia. It was first described by Kim and Szeto in 1937 and coined in 1948 by Kimura, a Japanese pathologist who detailed the pathological features of the disease (1). KD usually affects young men (male-to-female ratio, 4:1) in their second to third decade of life, and it is more common in East and Southeast Asia (2–4). KD etiology is unknown, but some associated risk factors are autoimmune diseases, allergy, cancer, and parasite infestation (5). The classic presentation of KD involves painless soft tissue swelling, mainly in the head and neck, as well as a single or multiple papules, nodules, and/or masses. Regional lymph nodes and salivary glands are usually affected, and the most common involved site is the periauricular area. Peripheral blood analysis reveals eosinophilia and an increase in serum IgE levels. The histologic aspect of lymph node in KD depicts angiolymphoid hyperplasia and intense eosinophilic infiltration (2, 5).

Kidney disease is the most common finding regarding systemic manifestations, affecting 15%–18% of adult patients with KD (3). Renal manifestation ranges from asymptomatic urinalysis abnormalities to chronic kidney disease. Proteinuria is reported in 12%–16% of patients with KD (6) and nephrotic syndrome is reported in approximately 60% of kidney involvement in KD (7). Kidney biopsy may present different histologic presentations such as membranous nephropathy (MN), IgA nephropathy (IgAN), minimal change disease (MCD), focal segmental glomerulosclerosis (FSGS), and membranoproliferative glomerulonephritis (MPGN) (3).

Herein, we report a case of overlapping MN and IgAN in a patient with KD.

## Case report

A 27-year-old Brazilian man was admitted complaining of increasing cervical mass and fever for the last 2 months, which had been recently associated with concomitant progressive bilateral leg edema. The patient denied fever, sweating, weight loss, contact with animals, use of medication, pruritus, or skin lesions. Physical examination upon admission revealed a patient with anasarca, as well as a bilateral cervical mass, more prominent on the left, with enlarged submental lymph nodes. The cervical mass was painless and there was no evidence of phlogistic signs. The spleen and liver were not palpable in the abdominal examination.

**Figure 1:**
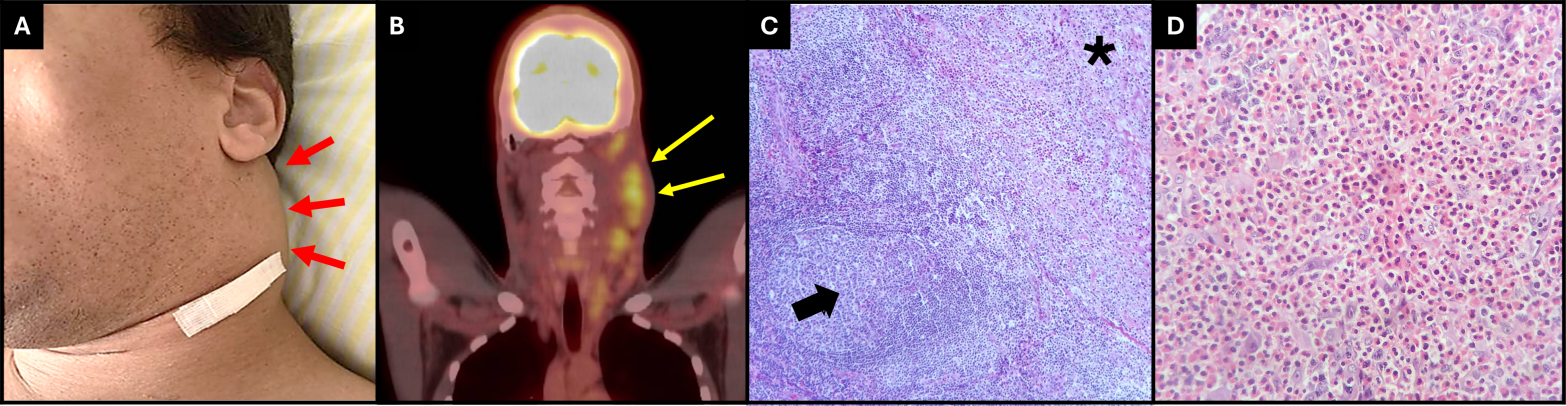
**(A)** Picture revealing the patient’s left neck mass (red arrows) **(B)** PET-CT scan reveals a high uptake of tracer in left cervical lymphonodes. (yellow arrows). **(C)** Lymph node showing zone of eosinophil-rich polimormorphic infiltrate, proliferation of reactive endothelium, and interstitial collagen deposition in a nodular fashion (black asterisk). The rest of the nodal parenchyma reveals numerous reactive lymphoid follicles (black arrow) (HE, 100×). **(D)** High magnification of the eosinophil-rich polymorphic infiltrate (HE, 400×).

A cervical computerized tomography (CT) evidenced multiple confluents and enlarged lymph nodes in the left cervical (**Figure 1A**) and supraclavicular areas. Those lymph nodes exhibited high tracer uptake in the PET-CT scan (**Figure 1B**).

Laboratory workup showed the following: hemoglobin, 14.4 g/dL; leukocyte, 10,080/mm³; neutrophils, 3,630/mm^3^; lymphocytes, 2,340/mm^3^; monocytes, 1,070/mm^3^; and eosinophils, 3,200/mm³. His serum creatinine was 0.83 mg/dL [estimated glomerular filtration rate (eGFR) by CKD-EPI: 140 mL/min per 1.73 m^2^], without electrolytic or acid–base disorders. Urinalysis revealed hematuria and proteinuria, with a 24-h proteinuria of 12.9 g. Metabolic investigation revealed the following: serum albumin, 1.3 g/dL; total cholesterol, 434 mg/dL; low-density lipoprotein, 320 mg/dL; high-density lipoprotein, 79 mg/dL; serum triglycerides, 177 mg/dL; IgE, 750 kU/L; IgA, 358 mg/dL; IgG, 324 mg/dL; and IgM, 73 mg/dL. The normal ranges of laboratory tests are presented in **Supplementary Table 1**. Serologies for hepatitis B, hepatitis C, HIV, and VDRL were all negative. Complement C3 and C4 levels were normal. No monoclonal protein was detected in blood or urine. Autoimmune panel (antinuclear antibody, rheumatoid factor, and antineutrophil cytoplasmic antibodies) was negative. Parasite infestation was ruled out. The patient presented IgG+ for cytomegalovirus and Epstein–Barr virus, and had negative serologies for Toxoplasma and rubeola. Serum anti-PLA2R was negative. Ultrasound evaluation showed kidneys with normal size and echographic aspects.

A cervical lymph node biopsy revealed proliferation of polymorphic cell consisting of plasma cells, mature lymphocytes (**Figure 1C**), and a dense infiltrate of eosinophils (**Figure 1D**). There was a proliferation of vascular structures at the periphery of the lymph node with dilated lumens, irregular contours, and thin walls, sometimes containing some red blood cells within it, with some smooth muscle fibers and lined by endothelium of flattened cells, without atypia. Immunohistochemistry was positive for CD15 in the dense eosinophil infiltrate, positive for CD3 and CD20 in rare lymphocytes, and negative for CD30, cytokeratins AE1/AE3, CD1a, and CD21. Bone marrow biopsy detected no proliferative process. The association of clinical and histological findings was consistent with KD.

**Figure 2:**
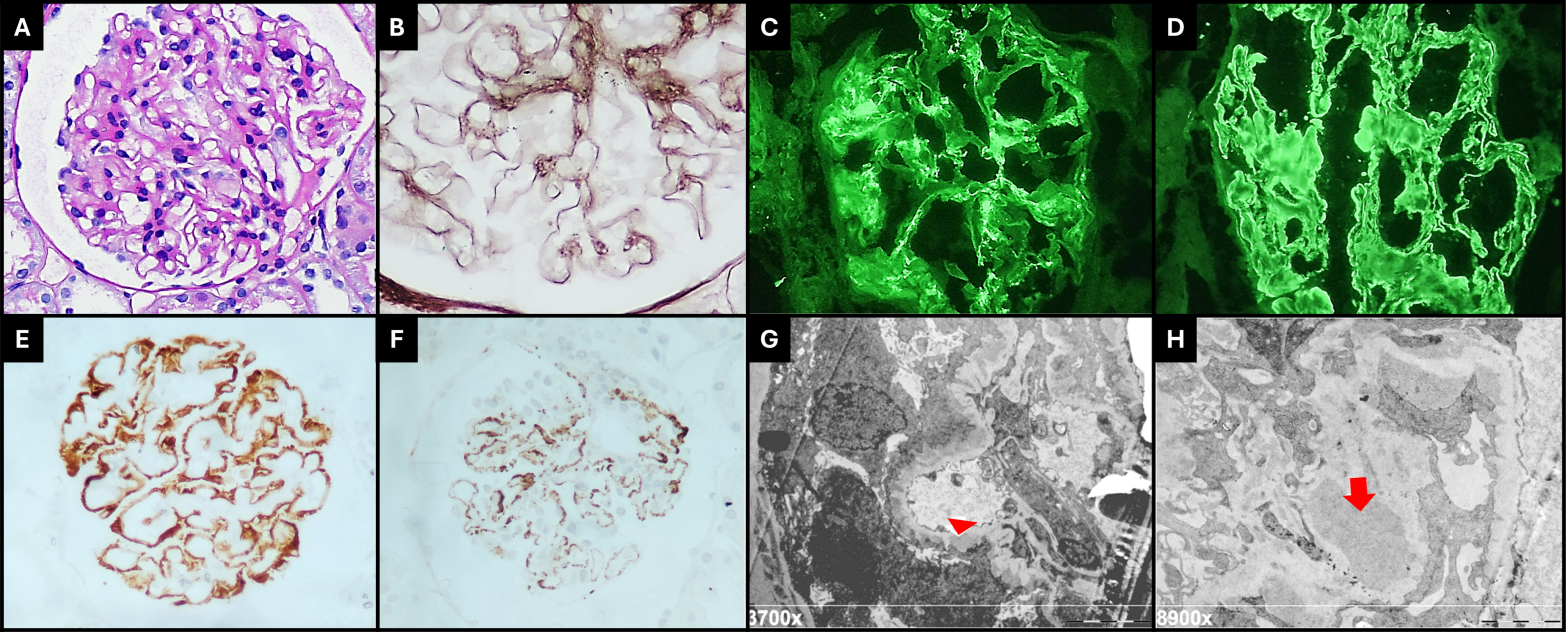
**(A)** Glomeruli show mild mesangial cell hipercellularity and matrix expansion (PAS, 400×). **(B)** The capillary walls are slightly thickened, and spikes are visible on Jones silver stain (400× + zoom). Immunofluorescence microscopy revealed diffuse granular deposits of IgA on mesangium and paramesangium [**(C)**, 400×], and IgG was diffusely positive on capillary walls, with a membranous pattern [**(D)**, 400×]. Immunohistochemistry staining for PLA2R [**(E)**, 400×] and IgG4 [**(F)**, 400×] were positive on capillary walls with a global and diffuse distribution. Electron microscopy shows subepithelial deposits (red arrow head) with surrounding basement membrane reaction, global podocyte foot process effacement [**(G)**, 3,700×], large electron-dense mesangial deposits (red arrow), and increased matrix [**(H)**, 8,900×].

A kidney biopsy was performed in the scenario of nephrotic syndrome. Light microscopy revealed the expansion of the mesangial matrix and glomerular basement membrane (**Figure 2A**) thickness with spike-like projections (**Figure 2B**). Immunofluorescence staining was global and diffuse positive for IgA (+2/+3) in the mesangium (**Figure 2C**), for IgG (+2/+3) (**Figure 2D**) and C3 (+2/+3) in capillary walls, and for kappa and lambda (+2/+3) in capillary walls and the mesangium. Immunohistochemistry highlighted a positive global and diffuse staining for PLA2R (**Figure 2E**) and IgG4 (**Figure 2F**) on capillary walls. Electron microscopy revealed electron-dense deposits in the subepithelial and mesangial region (**Figures 2G, H**). These findings were consistent with the overlapping of MN grade I/II associated with IgAN (MEST-C: M:0, E:0, S:0, T:0, C:0).

The patient was treated for KD with prednisone 1 mg/kg/d with progressive dose tapering and posterior association of methotrexate 15 mg/week. A renin–angiotensin system inhibitor was prescribed for nephrotic syndrome. The cervical mass reduced drastically, and the patient reached partial nephrotic syndrome remission. After a 3-year follow-up, his laboratory findings were as follows: Cr, 0.79 mg/dL; eGFR, 118 mL/min/1.73 m^2^; 24-h proteinuria, 1.8 g; serum albumin, 3.7 g/dL; and normal eosinophil and IgE levels.

## Discussion

KD is usually characterized by painless subcutaneous nodules in the head and neck. The diagnosis is confirmed by biopsy, and lymph node histology reveals angiolymphatic hyperplasia and intense eosinophilic infiltration. The pathogenesis of KD remains undetermined, but there is evidence of T-cell immunity dysregulation associated with allergy, parasite infections, vaccination, IgG4-related disease, and eosinophil and IgE disorders. Previous studies have shown that type 2 helper T (Th2) cytokines are elevated in patients with KD (1, 4, 8).

Kidney involvement is well documented in association with KD, especially nephrotic syndrome. Proteinuria usually appeared simultaneously with skin lesions, but in some cases, it can appear months or even years before (9). The pathogenesis of nephropathy seems to be related to alteration in the immune system, since nephrotic syndrome usually responds to immunosuppressive therapy (10). Another hypothesis is the existence of common etiological factors for both KD and glomerular lesion. Infectious agents or toxins may alter T-cell immunoregulation or induce an IgE-mediated type I hypersensitivity. This process results in the release of cytokines, which increases the permeability of glomerular basement membrane in glomeruli, causing proteinuria (3). Renal histology is variable and includes MN, IgAN, MCD, FSGS, and MPGN, with MN being the most common presentation (3, 11, 12). Other kidney disorders include interstitial lesions, such as eosinophil infiltration, and renal insufficiency (3, 11). We performed a narrative review from literature searching for the association of glomerular diseases and KD. **Table 1** summarizes clinical cases and treatment of KD associated with MN or IgAN.

**Table 1:** Glomerular diseases related to Kimura’s disease, treatment, and therapeutic response.

	Number of patients	Histologic diagnosis	Treatment	Response
Yang, 2019 (11)	12	MCD: 9 cases IgAN: 2 cases MN: 1 case	Steroids	8/10 Complete remission
Okura, 2014 (13)	1	MN: 1 case	Steroids	Complete remission
Natov, 1998 (9)	1	IgAN: 1 case	Steroids	Complete remission
Chen, 2016 (12)	29	Mesangium proliferation: 14 cases MCD: 8 cases FSGS: 3 cases MN: 2 cases MPGN: 1 case ATN: 1 case	*Tripterygium wilfordii*, Prednisone, Leflunomide, Tacrolimus, MMF, or Renin–angiotensin system blockers	22 complete remission, 4 partial remission
Yi, 2022 (14)	1	MN: 1 case	Steroids and tacrolimus	Complete remission
Obata, 2010 (15)	1	MN: 1 case	Steroids	Complete remission
Vissing-Uhre, 2021 (16)	1	MN: 1 case	Steroids Cyclosporine Cyclophosphamide Rituximab	Complete remission
Lee, 2014 (17)	1	MN: 1 case	Steroids Cyclosporine Surgical resection	Complete remission
Gaillard, 2017 (18)	1	MN: 1 case	Steroids Mycophenolate	Complete remission
Zhang, 2019 (19)	1	IgAN: 1 case	Steroids Cyclophosphamide	Complete remission
Liu, 2008 (20)	8	Mesangial proliferation with or without IgA deposition: 6 cases MN: 2 cases	Steroids	Complete remission
Hu, 2014 (21)	1	IgAN: 1 case	Steroids	Complete remission
Matsuda, 1992 (22)	2	MN: 1 case MCD: 1 case	Steroids	Complete remission

MCD, minimal change disease; IgAN, IgA nephropathy; MN, membranous nephropathy; FSGS, focal segmental glomerulosclerosis; MPGN, membranoproliferative glomerulonephritis; ATN, acute tubular necrosis; MMF, mycophenolate mofetil.

The present case illustrates an overlapping of MN and IgAN in a patient with KD. The occurrence of overlapping MN and IgA is rare in the literature and, to the best of our knowledge, has not been previously reported. IgAN is the most common primary glomerular disease worldwide, with a major prevalence in young patients. On the other hand, MN is more frequent in patients at age ≥40 years and may frequently be associated with systemic diseases. The main kidney manifestation of IgAN is hematuria, while MN is characterized by proteinuria and nephrotic syndrome. The histology of IgAN is characterized by IgA immunocomplex deposits in the mesangium, and proliferative forms occur in many cases. The histology of MN presents thickening of the glomerular basement membrane due to subepithelial granular IgG deposits associated with M-type phospholipase A2 receptor antibody (anti-PLA2R) in most primary subtype cases. The patients with overlap syndrome show features of both diseases (23). The incidence of MN–IgAN overlap is rare, with onset during middle age and predominantly affecting male patients. It is reported that MN–IgAN presents a higher proteinuria level, including nephrotic syndrome, compared to isolated IgA, and a similar (or less) frequency of nephrotic syndrome to MN. The galactose-deficient IgA1 levels are low in the MN–IgAN overlap or similar compared to IgAN and plasma anti-PLA2R positivity, which is less frequent than in MN (23–25).

A study on histological features showed that MN–IgAN exhibits significantly increased mesangial cell proliferation, matrix expansion, and inflammatory cell infiltration, with higher proportions of arteriole hyalinosis and lesions when compared to MN alone (26). MN–IgAN treatment in the literature is variable, which is similar to MN management, including renin–angiotensin system inhibitors for mild cases and corticosteroids plus cyclophosphamide or mycophenolate and/or calcineurin inhibitors for severe cases. The prognosis of MN–IgAN seems to be significantly better than IgAN, but outcomes have similar results when compared to MN (23–25).

MN is the most common histological pattern in patients with KD and proteinuria (3). Although it could be considered as secondary MN, there are reports with anti-PLA2R subepithelial deposition in these cases, typically of idiopathic MN (13). As MN is well documented in KD, it is difficult to consider two independent diseases occurring at the same time. KD seems to be associated with immunological disorder and MN is mediated by immunocomplexes. The production of antibodies against PLA2R antigens can be part of immune system dysregulation due to KD. On the other hand, the association between IgAN and KD is extremely rare, with few cases reported in the literature (9, 11). Considering that both KD and IgAN are more common in the Asian population, it would be possible to explain the independent occurrence of these diseases. However, the response of nephrotic syndrome and IgAN related to corticosteroids raises the hypothesis of immune dysregulation secondary to KD (9).

There is no consensus about the treatment for KD with kidney involvement. Corticosteroids have been the main and initial treatment for skin lesions and nephrotic syndrome. However, other immunosuppressive therapies are being used, including cyclosporin, mycophenolate, tacrolimus, rituximab, and cyclophosphamide. Other drugs have been used for KD treatment such as methotrexate and mepolizumab (27, 28). Irradiation and surgical removal have already been used for masses and subcutaneous nodules (3). Recurrence is common and proteinuria/nephrotic syndrome can relapse before or after skin lesions (9). Our case was managed with corticosteroids, renin–angiotensin system inhibition, and methotrexate with posterior prednisone dose reduction, progressing to partial response of proteinuria and complete cervical mass regression, without recurrence.

In conclusion, although uncommon, renal involvement as a glomerular disease must be considered in patients with KD.

## Data availability statement

The original contributions presented in the study are included in the article/**Supplementary Material**. Further inquiries can be directed to the corresponding author.

## Ethics statement

The studies involving humans were approved by Ethics Committee - Hospital Alemão Oswaldo Cruz. The studies were conducted in accordance with the local legislation and institutional requirements. The participants provided their written informed consent to participate in this study. Written informed consent was obtained from the participant/patient(s) for the publication of this case report.

## Author contributions

GB: Data curation, Writing – original draft. PN: Conceptualization, Investigation, Supervision, Writing – original draft. SM: Conceptualization, Formal analysis, Investigation, Supervision, Writing – review & editing. AA: Formal analysis, Investigation, Supervision, Writing – review & editing. LC: Investigation, Methodology, Writing – original draft. YM: Formal analysis, Methodology, Writing – original draft. VS: Investigation, Writing – review & editing. ÉO: Investigation, Writing – review & editing. LP: Investigation, Writing – review & editing. AB: Investigation, Writing – review & editing. MF: Investigation, Writing – review & editing. PC: Investigation, Supervision, Writing – review & editing. AC: Investigation, Supervision, Writing – review & editing.
