# Supplementary material for: Overlap of membranous nephropathy and IgA nephropathy in a patient with Kimura’s disease: a case report and literature review
Source: Front Immunol. 2024 Jul 12;15:1404954. doi: 10.3389/fimmu.2024.1404954 (PMC11272546; doi:10.3389/fimmu.2024.1404954)
Supplement: Supplementary file 1 [file Table_1.pdf]

SUPPLEMENTARY MATERIAL

Supplementary table 1. Normal ranges of laboratory tests

Laboratory Tests	Normal range
Hemoglobin	13.5 - 17.5g/dL
Leucocytes	3.500 - 10,500 /mm^3
Neutrophils	1.700 - 7,000 /mm3
Lymphocytes	900 - 2,900 /mm3
Eosinophyls	50 - 500 /mm3
Monocytes	300 - 900 /mm3
Platelets	150,000 - 450,000 /mm3
Creatinine	0.70 - 1.30 mg/dL
24h-proteinuria	<0,30 g/24 h
Serum albumin	3.5 - 5.2 g/dL
Total Cholesterol	< 200mg/dL
Low-density cholesterol	< 100mg/dL
High-density cholesterol	> 40mg/dL
Triglycerides	< 150mg/dL
IgE	< 100 kU/L
IgA	50 - 400 mg/dL
IgM	50 - 300 mg/dL
IgG	600 - 1,500 mg/dL
